# Mechanical Response and Shear-Induced Initiation Properties of PTFE/Al/MoO_3_ Reactive Composites

**DOI:** 10.3390/ma11071200

**Published:** 2018-07-12

**Authors:** Junyi Huang, Xiang Fang, Shuangzhang Wu, Li Yang, Zhongshen Yu, Yuchun Li

**Affiliations:** College of Field Engineering, PLA Army Engineering University, Nanjing 210007, China; huangjunyi357@163.com (J.H.); fangxiang3579@163.com (X.F.); wsz17512596128@sina.com (S.W.); 674670871yangli@sina.com (L.Y.); 13337720452@163.com (Z.Y.)

**Keywords:** mechanical response, PTFE/Al/MoO_3_ materials, split Hopkinson pressure bar (SHPB), constitutive model, shear-induced initiation

## Abstract

Polytetrafluoroethylene/aluminum/molybdenum oxide (PTFE/Al/MoO_3_) reactive composites of a volume ratio of 60:16:24 were studied in this research. Quasi-static compression, dynamic compression and drop-weight experiments were performed to explore the mechanical response and the shear-induced initiation properties of the composites. Mesoscale images of the specimens after sintering demonstrate that PTFE, Al and MoO_3_ powders were evenly mixed and no chemical reaction occurred after the materials were stirred, pressed and sintered. The yield stress and compressive strength of PTFE/Al/MoO_3_ specimens are sensitive to strain rate within the range of 10^−3^~3 × 10^3^ s^−1^, and the yield stress shows a bilinear dependence on the logarithm values of strain rate. The established Johnson-Cook constitutive model based on the experimental data can describe the mechanical response of PTFE/Al/MoO_3_ material well. Drop-weight tests show that the PTFE/Al/MoO_3_ specimens can react violently when impacted, with the characteristic drop height (*H*_50_) calculated as 51.57 cm. The recovered specimens show that the reaction started from the outer edge of the specimen with the largest shear force and the most concentrated shear deformation, indicating a shear-induced initiation mechanism. The reaction products of PTFE/Al/MoO_3_ specimens were AlF_3_, Al_2_O_3_, Mo and C, demonstrating that redox reaction occurred between PTFE and Al, and between Al and MoO_3_.

## 1. Introduction

Reactive materials (RMs) are a special type of energetic material, usually composed of at least two components which are not active on their own. These materials mainly consist of combinations of thermites, intermetallics, metal/polymer mixtures, metastable intermolecular composites (MICs), matrix materials or hydrides [1,2]. When subjected to external loads, RMs can undergo huge exothermic reactions, with plenty of chemical energy released. Of all the different types of RMs, mixtures of PTFE (polytetrafluoroethylene) and Al have been extensively studied, because of their large heat of reaction and the excellent combinations of performance that PTFE exhibits such as low friction coefficient, high thermal stability, high electrical resistance, high chemical inertness, high melting point and easiness of forming [3]. Therefore, many scholars have carried out extensive and in-depth researches on PTFE/Al materials. Raftenberg et al. [4] studied the deformation properties and compressive mechanical responses of solid rods of PTFE/Al composites with a mass ratio of 74:26, and the experimental data were obtained by conducted quasi-static Instron compression tests and split-Hopkinson pressure bar (SHPB) experiments. Based on these data, a fit to Johnson-Cook model was determined. In their studies, finite element simulation, using the Johnson-Cook model established above, has also been performed to compare with experimental values. Ge et al. [5] conducted two-dimensional microscale finite element analyses to study the mechanical behavior of PTFE/Al composites, and the real microstructure-based models and simulated microstructure models were both used. In order to improve the density and strength of the PTFE/Al materials, many researchers have tried to add tungsten (W) into the PTFE/Al composites. Cai et al. [3] performed dynamic compression tests in a drop-weight apparatus to explore the behavior of a PTFE/Al/W mixture at a high-strain and high-strain rate. They found that the initiation and propagation of cracks originate from the separation of the W particle-PTFE interface and PTFE nanofiber formed in specimens which were extensively deformed. The dynamic mechanical properties of PTFE/Al/W with different W particle sizes were also studied by Cai et al. [6], and they found that materials with fine particles have higher mechanical strength. Combining simulation and experiments, Herbold et al. [7,8] investigated the influence of particle size and sample porosity on the dynamic mechanical properties, including strength, failure and shock behavior, of the PTFE/Al/W granular composites. They discovered that force chains can be formed in the specimens with fine metallic particles under dynamic loading, which accounted for the increased strength of these specimens. Other scholars also carried out studies of the PTFE/Al/W composites from other perspectives using different techniques, including quasi-static and dynamic compression, scanning electron microscope (SEM), X-ray diffraction (XRD), and differential scanning calorimetry (DSC) [9,10,11,12,13].

However, in the above FTFE/Al/W experiments, no reaction between Al and W was observed, only a small amount of WC and W_2_C formed, which were activated by the high heat as a result of fluorination of Al [11]. In addition, literatures about the addition of metal oxides, such as CuO, MoO_3_ and Fe_2_O_3_, into the PTFE/Al are scarce. But the heat generated by the reaction between Al and metal oxides (thermites) cannot be neglected. Al/MoO_3_ is a kind of high energy density thermite, with a heat of reaction of 4.7 kJ/g and an adiabatic temperature of 3253 K, which is higher than that of other common thermites such as Al/CuO, Al/Fe_2_O_3_, Al/MnO_2_ and Al/Bi_2_O_3_ [14]. However, the Al/MoO_3_ mixture is difficult to form, and it is generally used as a powder for combustion. The addition of PTFE to Al/MoO_3_ can greatly increase the burning rate [15] and the PTFE/Al/MoO_3_ composites can be processed into a specific shape. Therefore, based on Al/MoO_3_, PTFE is used as a binder to prepare PTFE/Al/MoO_3_ multifunctional structural reactive materials, which have the dual properties of PTFE/Al and Al/MoO_3_. Furthermore, because PTFE is easy to form, the PTFE/Al/MoO_3_ materials also can be processed into a structural body with a certain strength, which greatly expands the applications of thermites.

In order to ensure the stability and safety of the reactive materials in the process of manufacturing, transportation, storage, and the reliability during applications, it is necessary to understand the mechanical response and reaction performance of the reactive materials under different loading conditions. Therefore, in this research, a kind of PTFE/Al/MoO_3_ reactive material was prepared using cold-pressing and sintering technology. Firstly, the micro-structures of the as received raw materials and sintered PTFE/Al/MoO_3_ specimens were analyzed by the scanning electron microscope (SEM). Then the quasi-static and dynamic mechanical properties of the specimens at room temperature were tested using a universal testing machine and split Hopkinson pressure bar (SHPB) system. Finally, a standard drop-weight apparatus combined with a high-speed camera was utilized to explore the impact sensitivity and shear-initiation process of the PTFE/Al/MoO_3_ composites.

## 2. Experimental

### 2.1. Specimens Preparation

**Figure 1:**
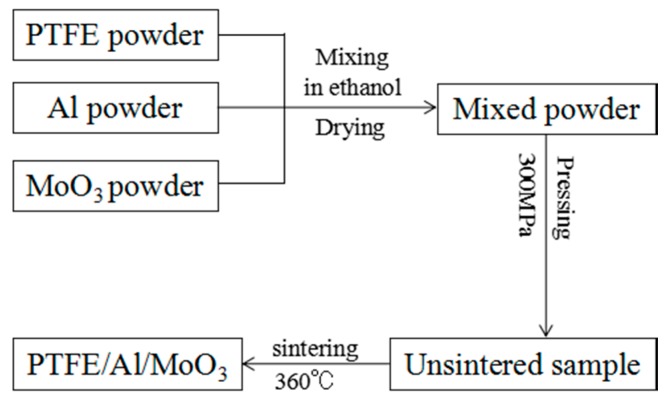
The preparation process of PTFE/Al/MoO_3_ specimens.

**Figure 2:**
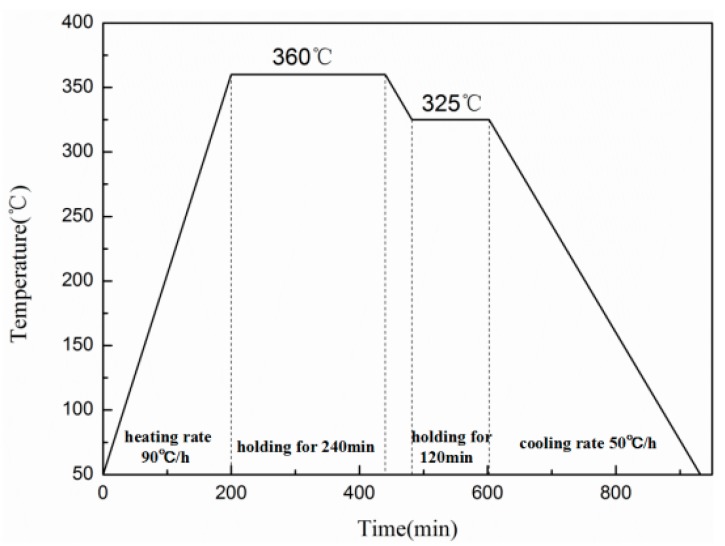
Sintering curve.

In this study, a kind of PTFE/Al/MoO_3_ composite was prepared with a volume ratio of 60:16:24. The ratio is based on the chemical equilibrium ratio of Al and MoO_3_, and PTFE is used as a binder. The original average sizes of the three raw materials were: PTFE: 25 μm (3M, Shanghai, China); Al: 1~2 μm (NAO, Shanghai, China); MoO_3_, 3 μm (NAO, Shanghai, China). The preparation process of the specimens can refer to Nielson’s work [16], which includes powder mixture, cold isostatic pressing and sintering, and is shown in Figure 1. The sizes of the specimens were Φ 10 mm × 10 mm, Φ 10 mm × 5 mm, and Φ 10 mm × 3 mm, which were used for the quasi-static compression, SHPB and drop-weight experiments, respectively. The properties of the specimens are greatly influenced by the sintering process [17], and the sintering process in this study is based on those described in [10,18,19]. The detailed process is as follows: The pressed specimens were put into a vacuum furnace, being heated from 25 °C to 360 °C at 90 °C/h, incubated for 240 min, then cooled to 325 °C at the rate of 50 °C/h, kept for 120 min, and finally cooled to 25 °C at the same rate. The sintering process curve is shown in Figure 2.

### 2.2. Quasi-Static Compression Experiments

The quasi-static compression experiments were carried out by the microcomputer-controlled universal testing machine (CMT5105, MTS, Eden Prairie, MN, USA) with a maximum load of 100 kN. The head of the testing machine travels downward at a speed of 0.6 mm/min, 6 mm/min and 60 mm/min, respectively, corresponding to the strain rate of the specimen of 0.001 s^−1^, 0.01 s^−1^ and 0.1 s^−1^. Before tests, all contact surfaces of the specimens were coated with vaseline to eliminate the effect of friction. Three specimens were tested under the same conditions to ensure the accuracy of the results.

### 2.3. SHPB Experiments

The dynamic compression properties of PTFE/Al/MoO_3_ specimens were tested using a split Hopkinson pressure bar (SHPB) system (AOC, Hefei, China). SHPB technology was originally proposed by Hopkinson [20] and later improved by Kolsky [21]. The SHPB technique is on the basis of two assumptions, namely (i) the one-dimensional stress wave assumption and (ii) the specimen is in force equilibrium and is deforming uniformly. The stress, strain, and strain rate in the specimen could be calculated as follows:

(1) $$\left\{ \begin{array}{l} \sigma_{e}=\frac{A_{0}}{A_{s}}E_{0}\varepsilon_{t}(t)\\ \varepsilon_{e}=-2\frac{C_{0}}{L_{s}}\int_{0}^{t}\varepsilon_{r}(t)dt\\ {\dot{\varepsilon }}_{e}=-2\frac{C_{0}}{L_{s}}\varepsilon_{r}(t) \end{array}\right.$$

where $\sigma_{e}$, $\varepsilon_{e}$, ${\dot{\varepsilon }}_{e}$ are the engineering stress, engineering strain and strain rate of the specimen, $\varepsilon_{r}(t)$ and $\varepsilon_{t}(t)$ are reflected and transmitted strain histories sensed by strain gages. *A*_0_ is the cross-sectional area of the compression bars, *E*_0_ is the Young’s modulus of the bars, *C*_0_ is the elastic wave velocity in the bars. *A_s_* and *L_s_* are the original cross-sectional area and length of the specimen.

**Figure 3:**
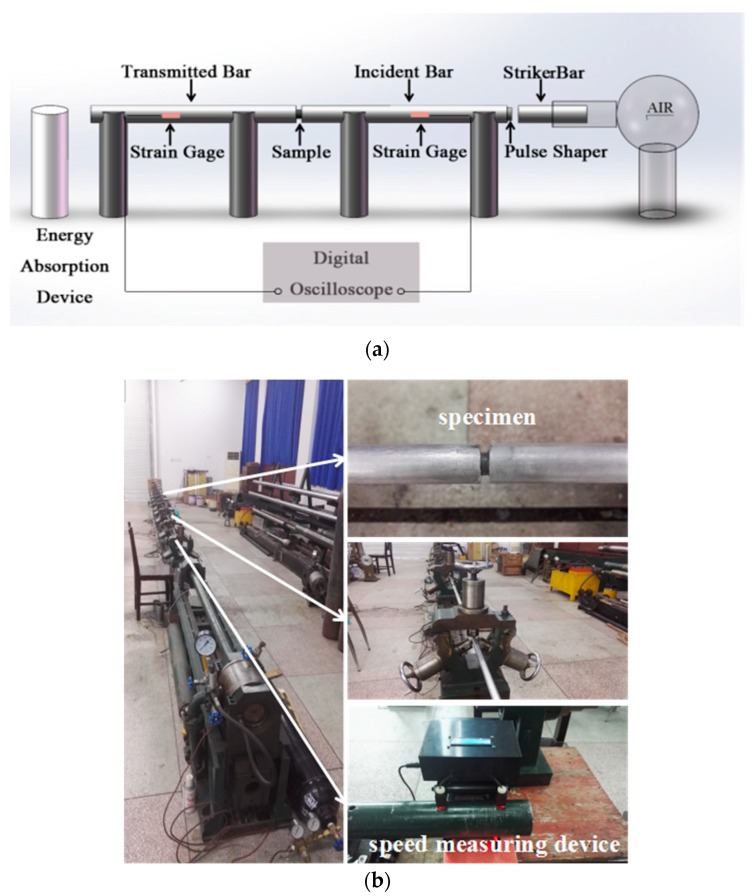
SHPB setup. (**a**) diagrammatic sketch; (**b**) actual device.

The diagrammatic sketch and actual device of the SHPB test system are given in Figure 3. The diameters of the striker bar, incident bar and transmitted bar are all 20 mm, and the lengths are 600 mm, 6000 mm, and 3500 mm, respectively. The distances of the strain gauges in the incident bar and the transmitted bar from the specimen are 3000 mm and 1300 mm, respectively. Because the mechanical impedance of PTFE/Al/MoO_3_ samples is relatively low, the signal in the transmitted bar is weak. So LC4 aluminum bars were used in the tests to improve the signal-to-noise ratio in the transmitted bar. If impedance-matched materials are used for the striker bar and the incident bar, the rise time of the generated pulse (possibly a square wave) will be short when the striker bar hits the incident bar, which is not conducive to facilitate stress-state equilibrium in the specimens. Therefore, during the experiments, a soft and deformable pulse shaper, such as rubber, paper, etc., can be placed on the incident end of the incident bar to increase the rise time of the generated wave. So in this study, a pulse shaper made of rubber with a thickness of 1 mm and a diameter of 10 mm was used.

### 2.4. Drop-Weight Experiments

**Figure 4:**
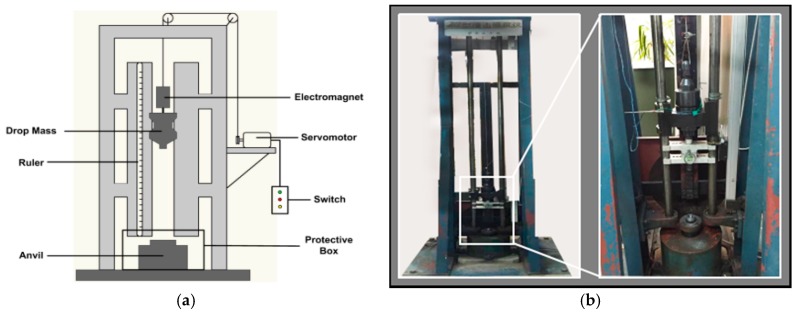
Drop-weight apparatus. (**a**) schematic illustration; (**b**) actual apparatus.

The impact sensitivity of the PTFE/Al/MoO_3_ material was investigated using a standard drop-weight apparatus in conjunction with a high-speed camera, and the schematic illustration and the actual apparatus are presented in Figure 4. The apparatus consists of a stainless steel plate (mass ca. 10 kg) which can be released from a height of 150 cm. When the drop mass was released, the high-speed camera was used to record the deformation process and reaction phenomenon of the specimen, and to determine whether the specimen was ignited based on the recorded images according to the “up-and-down technique” [22]. Once the suitable range of positive and negative reactions was found, the tests were performed at 2 cm intervals.

## 3. Results and Discussion

### 3.1. Mesoscale Characteristics

**Figure 5:**
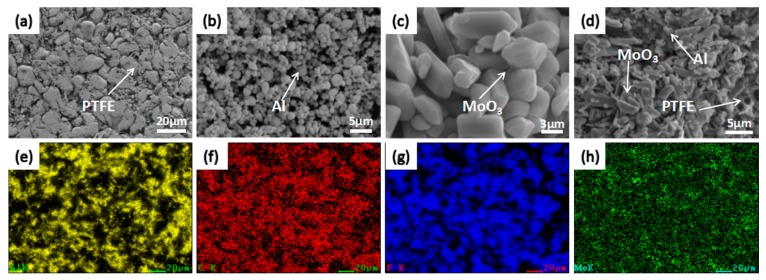
Microstructures and element distribution of the raw materials and a sintered specimen. (**a**) PTFE; (**b**) Al; (**c**) MoO_3_; (**d**) PTFE/Al/MoO_3_; (**e**) Al element; (**f**) C element; (**g**) F element; (**h**) Mo element.

The initial microstructures and element distribution of the raw materials and a sintered specimen were investigated by scanning electron microscope (HITACHI S-4800, Tokyo, Japan), as given in Figure 5. It can be seen from the Figure 5a–d that the PTFE is soft and irregular, with an average size of 20–25 μm, Al particles have a regular spherical shape with a particle size of 800 nm to 2 μm, and MoO_3_ particle is an irregular polyhedron with a smooth surface and a size of 2–5 μm. The PTFE/Al/MoO_3_ composite material is evenly mixed, with the Al and MoO_3_ particles in good contact and scattered in the PTFE matrix. The distribution of Al, C, F, and Mo element (Figure 5e–h) also confirmed the uniform mixing of PTFE, Al and MoO_3_ powders.

**Figure 6:**
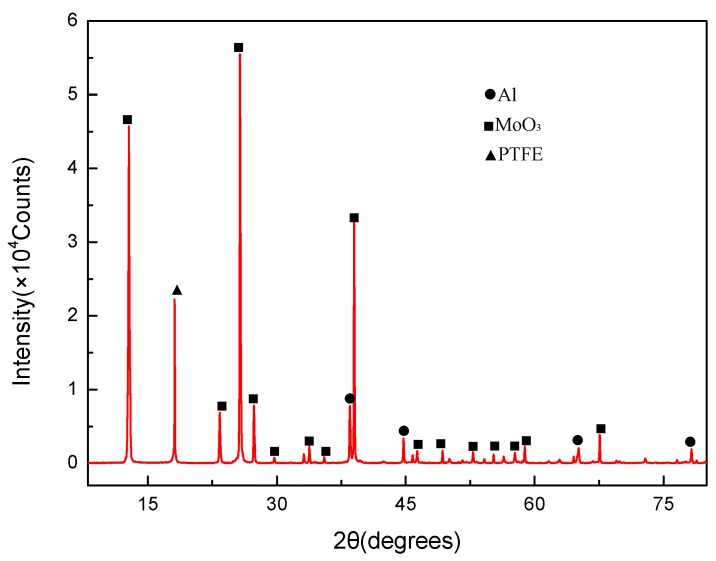
XRD pattern of PTFE/Al/MoO_3_ composite.

In the XRD pattern of the sintered PTFE/Al/MoO_3_ composite (Figure 6), only the diffraction peaks of PTFE, Al, and MoO_3_ were detected, indicating that no chemical reaction occurred after the material was stirred, pressed and sintered.

### 3.2. Mechanical Responses under Quasi-Static Compression

**Figure 7:**
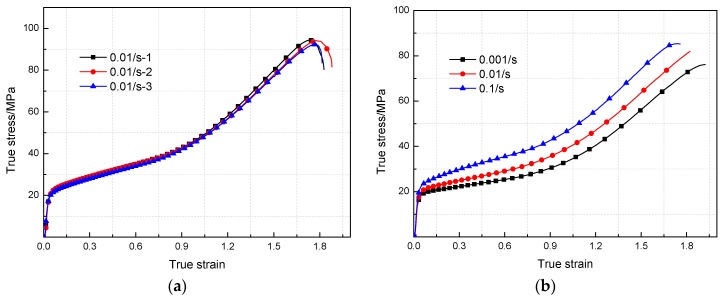
True stress-strain curves of PTFE/Al/MoO_3_ specimens under quasi-static compression. (**a**) Strain rate 10^−3^ s^−1^; (**b**) Strain rate 10^−3^~10^−1^ s^−1^.

Figure 7a presents the true stress-strain curves for three tests of PTFE/Al/MoO_3_ specimens at the strain rate of 0.01 s^−1^. The results imply that the three curves almost overlapped together, and the experimental error is 1.02%~3.17%, indicating that the experimental data in this study are repeatable and reliable.

The true stress-strain curves of PTFE/Al/MoO_3_ specimens under quasi-static compression at different strain rates are displayed in Figure 7b, and their corresponding parameters are summarized in Table 1. The data reveal that the yield stress and compressive strength of the specimens both present an increasing trend as the strain rate increases. But the failure strains show the opposite trend. However, the elastic moduli of PTFE/Al/MoO_3_ specimens remain almost unchanged, demonstrating that the strain rate has a limited influence on the elastic part of the specimens. PTFE/Al/MoO_3_ material is a typical particle-reinforced composite material whose mechanical properties mainly depend on the PTFE matrix and the interface formed between the matrix and the particles (Al and MoO_3_). In the initial stage of loading, the specimens showed linear elastic behavior, but the entire elastic deformation stage was short and the corresponding maximum strain was about 0.03. After yielding, the specimens experienced strain hardening. As the compressive load continued to increase, the molecular chains in the crystallization zone of the PTFE matrix began to slip, resulting in micro cracks, then the matrix and particles gradually “de-bonded”. When the load reached the maximum value, the specimens failed.

### 3.3. Dynamic Compression Performance

**Figure 8:**
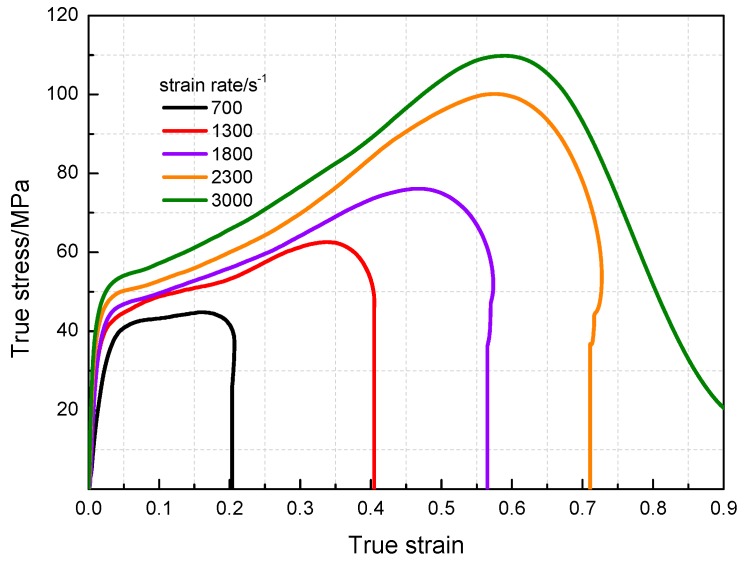
True stress-strain curves of PTFE/Al/MoO_3_ specimens under dynamic impact.

The compressive mechanical properties of PTFE/Al/MoO_3_ specimens at high strain rates were measured using SHPB system (AOC, Hefei, China). The true stress-strain curves are presented in Figure 8, and the corresponding dynamic mechanical properties parameters are shown in Table 2. As is shown in Figure 8, the specimens are strongly sensitive to the strain rates. Same as the values under quasi-static compression, the yield stress and compressive strength of PTFE/Al/MoO_3_ specimens under dynamic compression also have strain rate effects. However, in the elastic stage, the stress-strain curves of the specimens become steeper as the strain rate increases, that is, the elastic modulus is sensitive to the strain rate, which is different from that under quasi-static compression.

**Figure 9:**
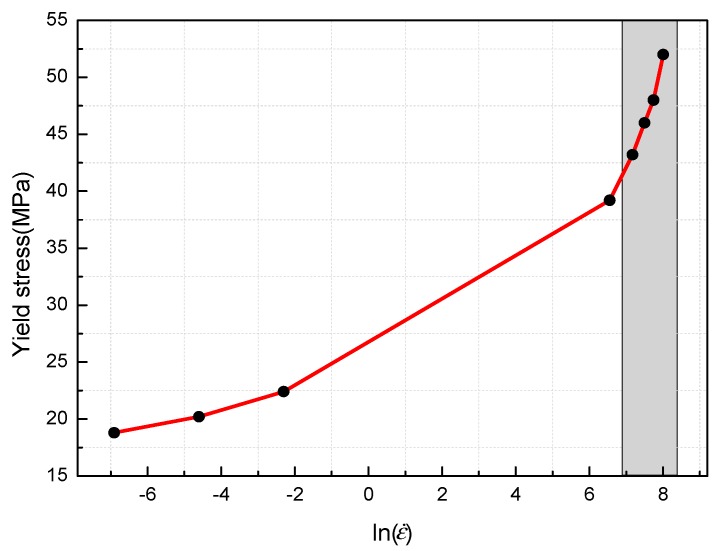
Relationship between yield stresses and logarithm train rates.

Figure 9 presents the relationship between the yield stresses of PTFE/Al/MoO_3_ specimens and logarithm values of strain rate. As can be seen from Figure 9, there is a clear bilinear relationship between the yield stresses and the logarithm values of strain rate. When the strain rate is greater than 10^3^ s^−1^, the slope of the curve suddenly increases. Walley et al. [18] also found this phenomenon when studying the mechanical properties of a series of polymers (including PTFE) at different strain rates. As previously discussed, the PTFE matrix is the main component of the PTFE/Al/MoO_3_ specimens, therefore, its mechanical properties have a great influence on the mechanical responses of PTFE/Al/MoO_3_ specimens.

**Figure 10:**
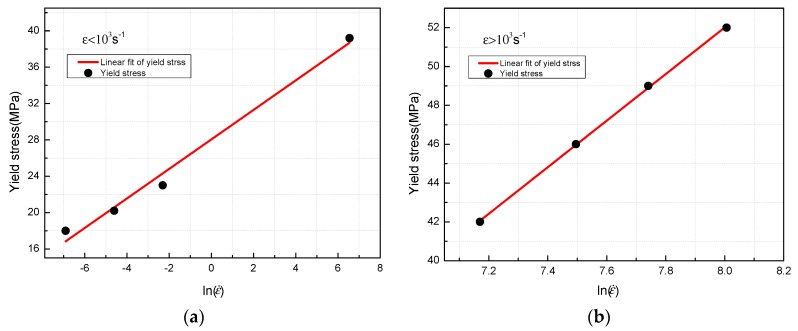
Linear fitting results of yield stresses and Logarithm values of strain rate. (**a**) $\dot{\varepsilon }<10^{3}\;s^{-1}$; (**b**) $\dot{\varepsilon }>10^{3}\;s^{-1}$.

A piecewise function can be used to describe the relationship between the logarithm values of strain rate and the yield stress, because there is a strain rate transition point at which the sensitivity of the yield stress to the strain rate changes greatly, as shown in Figure 9. The function can be expressed as:

(2) $$\left\{ \begin{array}{l} \sigma_{y}=1.62\ln(\dot{\varepsilon })+28,\;\dot{\varepsilon }<10^{3}\;s^{-1}\\ \sigma_{y}=12\ln(\dot{\varepsilon })-43.9,\;\dot{\varepsilon }\geq 10^{3}\;s^{-1} \end{array}\right.$$

where $\sigma_{y}$ is the yield stress, MPa; $\dot{\varepsilon }$ denotes strain rate, s^−1^. The correlation index R^2^ between the experimental values and the fitted equation is 0.98119 and 0.99939, respectively, indicating that the fitting results agree well with the experimental values. The linear fitting results of yield stresses and logarithm values of strain rate are shown in Figure 10.

### 3.4. Johnson-Cook Constitutive Model

The Johnson-Cook (JC) constitutive model is an empirical constitutive model based on a large number of practical applications. The data obtained in this study have been used to fit the JC constitutive equation. The specific details of the model are described in the researches of Johnson, Cook and Holmquist [23,24], and are briefly reviewed below.

(3) $$\sigma =\left(A+B\varepsilon_{p}{}^{n}\right)\left[1+\mathrm{Cln}(\dot{\varepsilon }/{\dot{\varepsilon }}_{0})\right](1-T^{*m})$$

where $\varepsilon_{p}$ denotes plastic strain, $\dot{\varepsilon }$ is the strain rate, ${\dot{\varepsilon }}_{0}$ is the reference strain rate. *A*, *B*, *n*, and *C* are all material constants. Since the tests in this study were all conducted at room temperature, regardless of the temperature softening effects of the material, so the JC equation is simplified to:

(4) $$\sigma =\left(A+B\varepsilon_{p}{}^{n}\right)\left[1+\mathrm{Cln}(\dot{\varepsilon }/{\dot{\varepsilon }}_{0})\right]$$

Figure 9 shows that there is a bilinear relationship between the logarithm values of strain rates and yield stresses of PTFE/Al/MoO_3_ specimens, and the critical strain rate is 10^3^ s^−1^. Therefore, the fitting process was divided into two parts, and the fitting parameters of the JC model are tabulated in Table 3.

From Table 3, the JC constitutive equation of PTFE/Al/MoO_3_ material is:

(5) $$\left\{ \begin{array}{l} \sigma =(18+10.23\varepsilon^{0.7169})(1+0.084\ln(\dot{\varepsilon }/{\dot{\varepsilon }}_{0})),\;\dot{\varepsilon }<10^{3}\;s^{-1}\\ \sigma =(43.2+77\varepsilon^{1.21353})(1+0.2255\ln(\dot{\varepsilon }/{\dot{\varepsilon }}_{0})),\;\dot{\varepsilon }\geq 10^{3}\;s^{-1} \end{array}\right.$$

**Figure 11:**
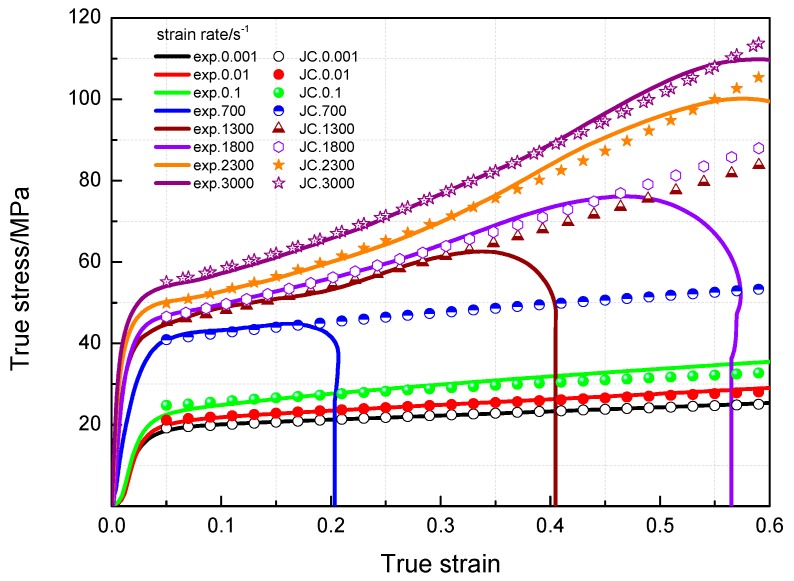
Comparison of JC model to experimental data at different strain rates.

The comparison of the fitting results of the JC model and experimental values at different strain rates for PTFE/Al/MoO_3_ specimens is presented in Figure 11. It can be drawn that the fitting results are in good agreement with the measured values. The established JC constitutive model can describe the mechanical response of PTFE/Al/MoO_3_ material well and can provide certain references for the practical applications of this material.

### 3.5. Drop-Weight Tests

**Figure 12:**
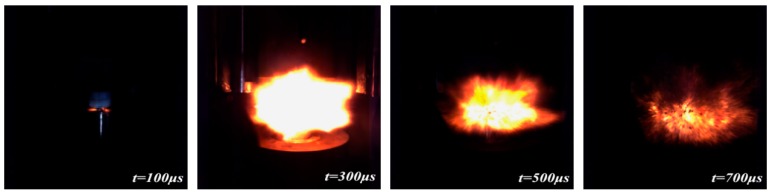
Reaction process of PTFE/Al/MoO_3_ specimens under drop-weight tests.

Figure 12 shows a sequence of images taken from a high-speed camera of the PTFE/Al/MoO_3_ specimens under drop-weight tests (drop height is 1.5 m). PTFE/Al/MoO_3_ specimens can react violently under the impact of a drop hammer, with intense light and a huge explosion sound. Moreover, the ignition time is about 100 μs and the burning of specimen lasts approximately 700 μs.

**Figure 13:**
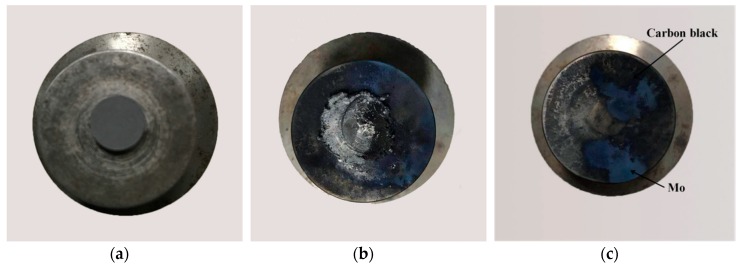
Typical photographs of the PTFE/Al/MoO_3_ specimens. (**a**) before reaction; (**b**) and (**c**) after reaction.

Unlike the reaction phenomenon of PTFE/Al, strong blue smoke was observed when the PTFE/Al/MoO_3_ specimens were impacted by the drop mass, which is probably due to the reaction of Al with MoO_3_, while black smoke was found when PTFE/Al reacted [25]. The typical photographs of the PTFE/Al/MoO_3_ specimens before reaction and after reaction are shown in Figure 13. From the recovered specimen (see Figure 13b), it can be seen that the reaction started from the outer edge of the specimen with the largest shear force and the most concentrated shear deformation [26], which demonstrates a shear-induced initiation mechanism. Ames [27], Lee [28] and Feng et al. [29] also observed a similar reaction mode when studying the reaction behaviors of PTFE/Al reactive materials. The reaction then propagated from the edge to the center of the specimens and quenched. We attribute the quenching to the rapid densification caused by the drop mass, because the confined space would limit the further shear deformation in the center of the specimens.

**Figure 14:**
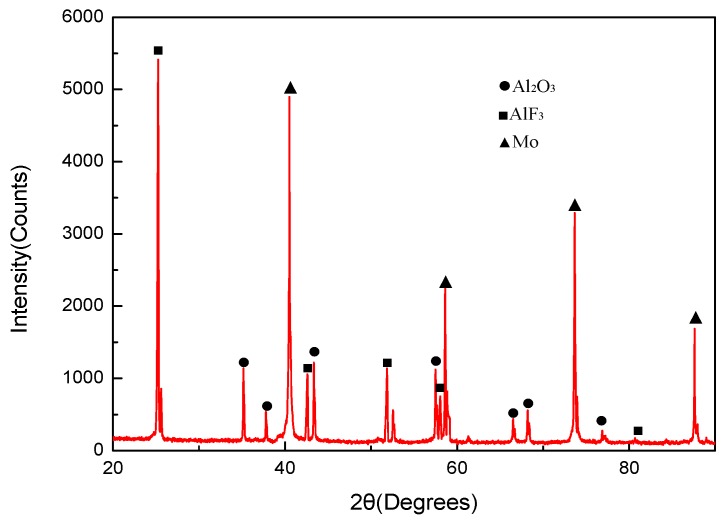
Reaction products of PTFE/Al/MoO_3_ after drop-weight tests.

Residues after reactions were collected and analyzed by XRD. Figure 14 presents the XRD pattern of the reaction products of PTFE/Al/MoO_3_ specimens. It can be concluded from the figure that the diffraction peaks of AlF_3_, Al_2_O_3_, and Mo were detected in the products. Moreover, carbon black (C) is also one of the reaction products and was not detected in the figure because of its amorphous phase. Therefore, it is probable that the initial chemical reactions of the specimens are triggered by PTFE and Al composites, followed by the reaction of Al and MoO_3_. Granier and Pantoya [30] found that the burning velocity of Al/MoO_3_ thermite is strongly influenced by the preheat temperature of the reactants. The adiabatic reaction temperature between PTFE and Al can even reach 4000 K [31], which can greatly promote the reaction of Al and MoO_3_. Furthermore, the state of the product metal (Mo) is also important for the reaction. At the high temperature produced by the reaction of PTFE and Al, Mo exists in liquid form, which will also facilitate the interaction between Al and MoO_3_ [32]. Combining the analysis above, the possible chemical reactions process of PTFE/Al/MoO_3_ can be described as:

4Al + 3(−C_2_F_4_−) → 4AlF_3_ + 6C (6)

2Al + MoO_3_ → Mo + Al_2_O_3_ (7)

**Figure 15:**
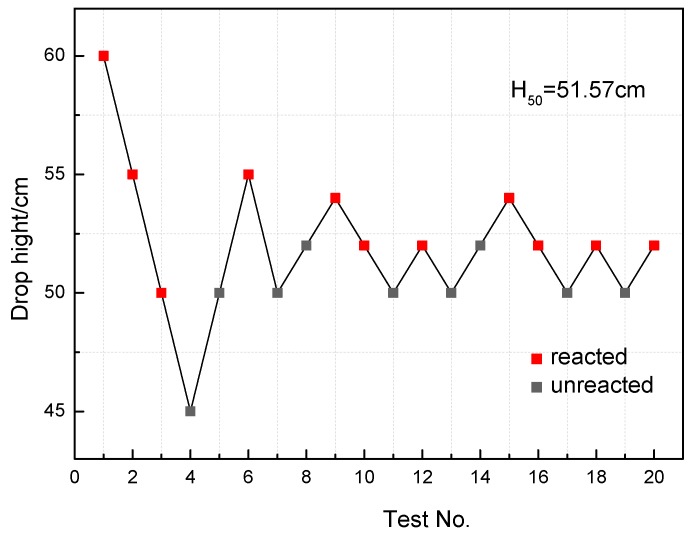
The drop-weight tests data points.

The sensitivity of PTFE/Al/MoO_3_ specimens was measured by the characteristic drop height (*H*_50_), when the drop mass is released from this height, the specimen has a 50% probability of ignition. *H*_50_ is calculated by the following formula:

(8) $$H_{50}=[A+B(\frac{\sum iC_{i}}{D}-\frac{1}{2})]$$

where *A* is the lowest height in the tests, *B* is the height increment, *D* is the number of reaction specimens, *i* is the order of the drop height, and *C_i_* is the number of reaction specimens at the specified height. The drop-weight tests were performed on 20 PTFE/Al/MoO_3_ specimens. Figure 15 shows the experimental results recorded according to the “up-and-down” methods [21]. The characteristic drop height of the PTFE/Al/MoO_3_ specimen was calculated as 51.57 cm according to Equation (8).

## 4. Conclusions

Quasi-static compression, dynamic compression and drop-weight experiments were performed to study PTFE/Al/MoO_3_ reactive composites prepared by cold-pressing and sintering technology. The conclusions can be drawn as follows:

(1) Mesoscale images of the specimens after sintering obtained by SEM demonstrate that PTFE, Al, and MoO_3_ particles were evenly mixed. And no chemical reaction occurred after the material was stirred, pressed and sintered.
(2) The yield stress and compressive strength of PTFE/Al/MoO_3_ specimens are sensitive to strain rate within the range of 10^−3^~3 × 10^3^ s^−1^. The elastic modulus is insensitive to the strain rate at low strain rate, but shows significant strain rate dependence at high strain rate.
(3) The yield stress shows a bilinear dependence on the logarithm values of strain rate, and the slope has an increase at the strain rate of 10^3^ s^−1^. The established JC constitutive model can describe the mechanical response of PTFE/Al/MoO_3_ material well and can provide certain references for the practical applications of this material.
(4) The recovered specimens show that the reaction started from the outer edge of the specimen with the most concentrated shear deformation, indicating a shear-induced initiation mechanism. The characteristic drop height of impact sensitivity (*H*_50_) of the PTFE/Al/MoO_3_ specimens was calculated as 51.57 cm.
(5) The reaction products of PTFE/Al/MoO_3_ specimens were AlF_3_, Al_2_O_3_, Mo and C, indicating that redox reaction occurred between PTFE and Al, and between Al and MoO_3_.

## Figures and Tables

**Table 1:** Mechanical properties parameters of PTFE/Al/MoO_3_ specimens. (under quasi-static compression).

Strain Rate/s^−1^	Elastic Modulus/MPa	Yield Stress/MPa	Compression Strength/MPa	Failure Strain
0.001	851	18	76	1.91
0.01	852	21	82	1.82
0.1	854	24	85	1.75

**Table 2:** Dynamic performance parameters of PTFE/Al/MoO_3_. (specimens at different strain rates).

Strain Rate/s^−1^	Elastic Modulus/MPa	Yield Stress/MPa	Compression Strength/MPa	Critical Strain
700	1226	39.2	44.9	0.17
1300	1456	43.2	62.8	0.35
1800	1895	46	76.3	0.49
2300	2568	48	100.4	0.59
3000	3569	52	110.2	0.60

**Table 3:** Fitting parameters of the JC model.

	$\dot{\varepsilon }<10^{3}\;s^{-1},\;{\dot{\varepsilon }}_{0}=10^{-3}\;s^{-1}$			$\dot{\varepsilon }>10^{3}\;s^{-1},\;{\dot{\varepsilon }}_{0}=1300\;s^{-1}$		
	Fitting Values	Standard Error	R^2^	Fitting Values	Standard Error	R^2^
A	18	0	1	43.2	0	1
B	10.2354	0.00492	0.99633	77.0012	0.0062	0.96842
n	0.7107	0.00387	0.99633	1.21353	0.00326	0.96842
C	0.084	0.12302	0.98039	0.2255	0.71825	0.98387
